# Stimulus dependent transformations between synaptic and spiking receptive fields in auditory cortex

**DOI:** 10.1038/s41467-020-14835-7

**Published:** 2020-02-27

**Authors:** Kyunghee X. Kim, Craig A. Atencio, Christoph E. Schreiner

**Affiliations:** 1 Coleman Memorial Laboratory, Department of Otolaryngology - Head and Neck Surgery, University of California San Francisco, San Francisco, USA; 2 Center for Integrative Neuroscience, University of California San Francisco, San Francisco, USA

**Keywords:** Neuroscience, Auditory system, Cortex, Cellular neuroscience, Neural circuits

## Abstract

Auditory cortex neurons nonlinearly integrate synaptic inputs from the thalamus and cortex, and generate spiking outputs for simple and complex sounds. Directly comparing synaptic and spiking activity can determine whether this input-output transformation is stimulus-dependent. We employ in vivo whole-cell recordings in the mouse primary auditory cortex, using pure tones and broadband dynamic moving ripple stimuli, to examine properties of functional integration in tonal (TRFs) and spectrotemporal (STRFs) receptive fields. Spectral tuning in STRFs derived from synaptic, subthreshold and spiking responses proves to be substantially more selective than for TRFs. We describe diverse spectral and temporal modulation preferences and distinct nonlinearities, and their modifications between the input and output stages of neural processing. These results characterize specific processing differences at the level of synaptic convergence, integration and spike generation resulting in stimulus-dependent transformation patterns in the primary auditory cortex.

## Abstract (web summary)

The authors compare receptive fields and nonlinearities of synaptic inputs, membrane potentials, and spiking activity in the auditory cortex for broadband stimuli revealing distinct differences, which lead to an increase in feature selectivity from neuron input to output. Frequency selectivity is distinctly higher for spectrotemporal receptive fields (STRFs) than for tonal receptive fields (TRFs).

## Introduction

Auditory cortical circuitry shapes spectral processing by nonlinearly integrating converging auditory information across frequency and time. Synaptic inputs are integrated and subsequently transformed into a spiking output. As a consequence, cortical spectral tuning properties, such as bandwidth of tonal receptive fields (TRFs), can differ from their inputs, widely vary, and demonstrate nonlinearly facilitated or suppressed responses (e.g., in two-tone stimuli^1–4^). Thus, spectral integration and the cellular transformation of information of more complex natural or dynamically modulated artificial sounds should be affected by these nonlinear processes. This suggests that receptive fields derived from complex sounds could differ significantly from those derived by combining single pure tone responses.

Auditory cortex neurons exhibit diverse and dynamic receptive fields in response to dynamically changing stimuli^5^. Synthetic stimuli that contain essential properties of natural sounds are effective tools for estimating the response properties of auditory cortex neurons because they are under full experimental control and can be modified to allow for the analysis of nonlinear response features. The dynamic moving ripple (DMR) is a complex sound that contains the essential modulation features common to many natural sounds^6^. Unlike many natural sounds, which are often non-Gaussian^7^, the DMR is globally uncorrelated. This aspect of DMRs supports rigorous estimates of receptive fields and associated nonlinear input–output functions by event-triggered receptive field estimation^8^.

Nonlinear interactions between stimulus elements preclude the use of certain methodologies to estimate the degree of spectral integration by quantifying receptive field features such as the spectral bandwidth. In the auditory cortex, spectral bandwidths for subthreshold responses have been examined predominantly for TRFs^9–12^. TRF bandwidths for subthreshold responses were found to be slightly broader than TRF bandwidths obtained from spikes^13,14^. This indicates that the subthreshold convergence of various excitatory and inhibitory inputs can be further refined by the spike-generation process. The mechanisms that underlie subthreshold and suprathreshold difference in TRF bandwidths between responses may not apply directly to more complex sounds. However, very few studies have related suprathreshold and subthreshold integration for stimuli with different sound statistics^15^.

The main goal of this study is to apply a quantitative, comparative approach to the different stages of information transformation at the neuronal level. For that purpose, we examine spectral and temporal tuning by comparing the suprathreshold and subthreshold receptive fields with in vivo whole-cell recordings using the blind patching approach. We find that the spectral tuning of spectrotemporal receptive fields (STRFs) in both subthreshold and suprathreshold responses is often much narrower than that of TRFs. The nonlinearities associated with sub- and suprathreshold STRFs reveal distinct differences. This suggests that spectral tuning in the primary auditory cortex (A1) is determined by different underlying influences when processing pure tones and complex stimuli. Furthermore, the best spectral and temporal modulation frequencies in STRFs from small subthreshold events are usually higher than for large subthreshold responses and spikes, suggesting that A1 neurons receive diverse inputs with respect to the modulation preferences that shape their output patterns. Examination of synaptic events underlying the generation of post-synaptic potentials (PSPs) reveals clear distinctions in excitatory and inhibitory STRFs further constraining the information transformation in A1.

## Results

### Tonal receptive fields

**Fig. 1:**
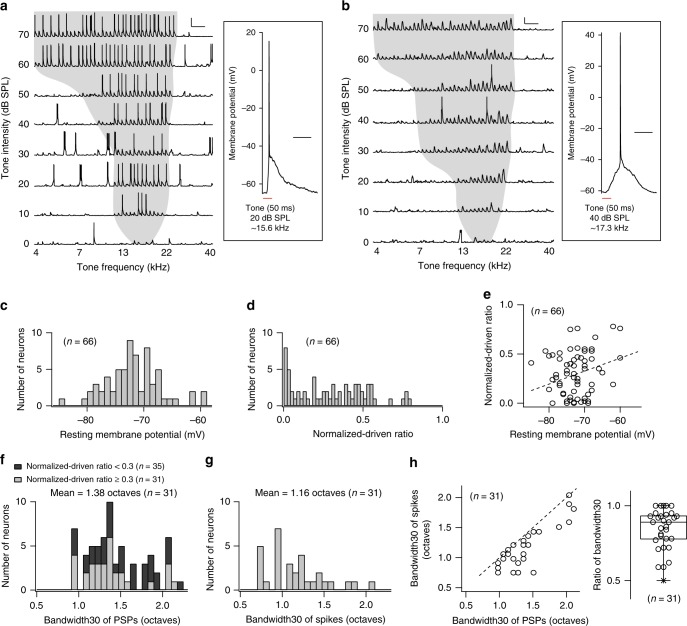
Tonal receptive fields obtained from in vivo current-clamp whole-cell recordings to pure tones. **a** A representative example with a high normalized-driven ratio of 0.57. This example had a characteristic frequency of ~17 kHz and a bandwidth30 of ~1 octave within a TRF region (the shaded area). Horizontal scale: 1 s; vertical scale: 30 mV. The inset on the right shows an enlarged view of one representative trace obtained from a tone among a set of 360 combinations. Scale bar, 0.1 s. **b** A representative example with a low normalized-driven ratio of 0.02. The neuron had a characteristic frequency of ~17 kHz and a bandwidth30 of ~1.7 octaves within a TRF region. Spikes have been truncated to illustrate relatively small subthreshold membrane potential responses well. Horizontal scale: 1 s; vertical scale: 10 mV. The inset on the right shows an enlarged view of one representative trace obtained from a tone among a set of 360 combinations. Scale bar, 0.1 s. **c** Histogram of resting membrane potentials (*n* = 66 neurons from 41 mice). **d** Histogram of normalized-driven ratios (*n* = 66). **e** Relationship between normalized-driven ratios and resting membrane potentials. **f** Histograms of the bandwidth30s of PSPs (normalized-driven ratio < 0.3, *n* = 35, dark gray; normalized-driven ratio ≥0.3, *n* = 31, light gray). **g** Histogram of the bandwidth30s of spikes (*n* = 31/66 with normalized-driven ratio ≥0.3). **h** Relationship between the bandwidth30s of PSPs and the bandwidth30s of spikes (*n* = 31/66 with normalized-driven ratio ≥ 0.3). The two groups were statistically significantly different (two-tailed paired Student’s *t*-test; *p* = 10^−6^). On the right, the box plot indicates ratios of the spike bandwidth30 to the PSP bandwidth30. The lower and upper hinges are at the 25th and 75th percentiles. The median by the middle line between hinges was 0.89. Asterisks indicate outliers. The minimum value is marked by the lower whisker.

We studied the responses of A1 neurons to pure tones and dynamic broadband stimuli estimating both TRFs and STRFs. Recordings were obtained largely at depths corresponding to layer 4, the main hub receiving lemniscal thalamic inputs from the ventral medial geniculate body^16^. Tone-evoked membrane potentials (e.g., Fig. 1a, b; *n* = 66) typically resulted in V-shaped TRFs for subthreshold PSPs, with a distinguishing trough at the minimum sound level needed to evoke a response and increasing bandwidth with increasing stimulus intensity. PSPs for tonal responses were identified based on their onset latencies (5–50 ms) relative to tone onset and response magnitudes (>4 × standard deviation above baseline). For pure tones the maximum PSP amplitude was 16.3 ± 4.8 mV (mean ± s.d., *n* = 66) (PSP rise time (10 − 90%): 30 ± 17 ms (mean ± s.d., *n* = 66); PSP decay time (90 − 10%): 86 ± 20 ms (mean ± s.d., *n* = 66)). Some recordings showed a high responsiveness where almost every PSP generated spikes (Fig. 1a), whereas others had only a few spikes (Fig. 1b) thus limiting the ability to obtain a spike-based TRF sufficiently reliable to estimate the spectral bandwidth. Therefore, we quantified the ratio of the number of spikes to the number of PSPs as the normalized-driven ratio (see the “Methods” section). The distribution of normalized-driven ratios resembled an exponential decay with a larger number of neurons near 0 and very few near 1 (Fig. 1d). The range of encountered resting membrane potentials (−85 to −59 mV) was consistent with previous observations^9,11^ (Fig. 1c). Normalized-driven ratios and resting membrane potentials were weakly correlated (*n* = 66; Pearson’s *r* = 0.18, *p* = 0.15) with more depolarized membrane potentials tending to produce more spikes (Fig. 1e). TRFs with normalized-driven ratio ≥0.3 yielded estimates of bandwidths at 30 dB above the minimum threshold (bandwidth30; see the “Methods” section) that were consistent with estimates at slightly higher or lower sound intensities. The average bandwidth30 from tonal PSPs with normalized-driven ratio ≥0.3 was 1.38 ± 0.33 octaves (Fig. 1f; mean ± s.d., *n* = 31). The corresponding bandwidth30 from spiking TRFs with normalized-driven ratio ≥0.3 was 1.16 ± 0.34 octaves (Fig. 1g; mean ± s.d., *n* = 31), indicating a narrower tuning for the spike bandwidth. Pairwise comparison of TRF bandwidth30s of PSPs and spikes (Fig. 1h, left) confirmed a narrowing of spiking versus PSP bandwidth with a median ratio ((bandwidth (spike))/(bandwidth (PSP))) = 0.9 (Fig. 1h, right; two-tailed paired Student’s *t*-test, *p* = 5 × 10^−7^). This result is consistent with previous intracellular estimates based on population analysis^13,14,17,18^. Thus, for the targeted best-frequency range, the average spiking bandwidth30 for TRFs was ~16% narrower than for subthreshold bandwidths. This reflects a systematic but moderate transformation of spectral selectivity between the input and output of single A1 neurons for pure tones.

### Spectrotemporal receptive fields

Tone-evoked responses are characterized by highly synchronous synaptic inputs at the onset of the tones. Since naturalistic stimuli are usually more sustained, have broader bandwidth, and contain spectro-temporal dynamics, it is essential to understand the differences between synaptic and spiking responses for this broad class of stimuli. Cortical neurons have robust and consistent spiking activity over the full duration of DMR stimuli^19–21^. Subthreshold, non-spike-related events also produced STRFs with stimulus-related features that were consistent across multiple measurements (Supplementary Fig. 2). In the case of DMR responses, PSPs are defined as membrane voltage fluctuations with a discernable peak (>4 × standard deviation above baseline; see the “Methods” section).

**Fig. 2:**
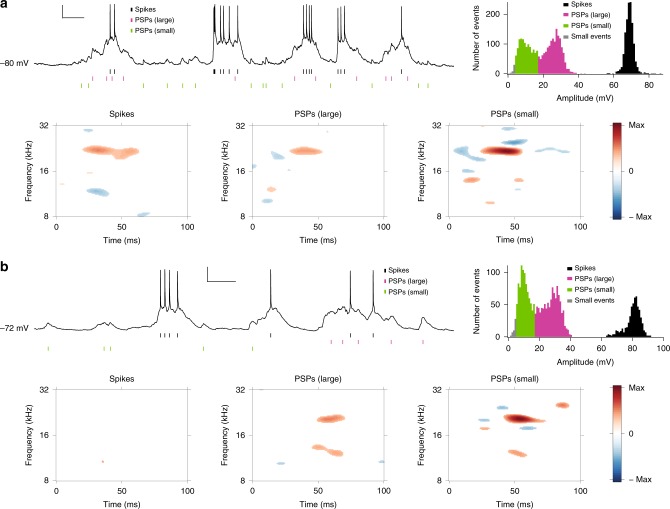
Spectrotemporal receptive fields obtained from in vivo current-clamp whole-cell recordings to the DMR stimulus. **a** Top left, a segment of a neuron response to the DMR stimulus. The short black line marks indicate spike times. Horizontal scale: 0.3 s; vertical scale: 15 mV. The magenta and green marks indicate peak times of PSPs with large and small amplitudes, respectively. Top right, an amplitude histogram of spikes and PSPs was obtained from the left recording and shows a bimodal PSP peak amplitude distribution. Bottom, the spike STRF (first), the STRF for large PSPs (second), and the STRF for small PSPs (third) resulted from spike times (black in the histogram), peak times of large PSPs (magenta in the histogram), and peak times of small PSPs (green in the histogram), respectively. The number of peaks included in computing each STRF was 1226 for spikes, 1437 for large PSPs, and 1162 for small PSPs. Bottom right, the color bar goes from the overall maximum (red) to the minus maximum (blue) on the same absolute scale of three STRFs from spikes, large PSPs, and small PSPs. **b** As in (**a**), but from a different neuron. Top left, horizontal scale: 0.3 s; vertical scale: 20 mV. Top right, an amplitude histogram shows a bimodal PSP peak amplitude distribution. The number of peaks included in computing each STRF (bottom) was 532 for spikes, 1009 for large PSPs, and 863 for small PSPs.

Recorded voltage traces show PSPs associated with spiking events as well as PSPs of various amplitudes not resulting in a spike. Peak-amplitude histograms represent spiking events with high values (Fig. 2a, b, top right; black). PSP peak amplitude distributions for non-spiking events were either bimodal (Fig. 2a, b, top right; magenta and green) or unimodal in 60% (*n* = 24/40) and 40% (*n* = 16/40) of the recorded neurons, respectively. For bimodal PSP histograms, PSP amplitudes were subdivided into large (magenta) and small (green) events at the trough between the two maxima. When a PSP histogram was unimodal, large and small PSP amplitudes were divided at approximately 50% of the number of non-spiking PSPs. Both large and small PSPs (Fig. 2) likely represent the integration of multiple synchronous synaptic inputs from many synapses, since unitary synaptic inputs usually have an amplitude of ~1 mV^22,23^.

STRFs were estimated for the three different response events by extracting peak timing information for spikes (black tick marks below the voltage traces), and non-spike related large (magenta), and small (green) PSPs (Fig. 2a, b, bottom). Very small events (marked gray in amplitude histogram) indicate events less than 4 × s.d. of unresponsive baseline segments and were excluded from further analysis. Most neurons (75%, *n* = 30/40) showed significant STRF subfields for spiking, large PSP, and small PSP events (Fig. 2a, bottom; example with a best frequency of ~21.7 kHz and a significant STRF bandwidth of ~0.28 octaves). For these neurons, the mean amplitudes from large PSPs ranged from 13.8 to 28.8 mV and the mean amplitudes from small PSPs were between 7.2 and 11.5 mV. The two amplitude ranges did not overlap. A second group (25%, *n* = 10/40) lacked significant spike-based STRF subfields but showed significant PSP-based STRF subfields (Fig. 2b, bottom; example with two peak frequencies at ~12.1 and ~20.2 kHz). This grouping was independent of the uni- or bimodal nature of the PSP peak amplitude distributions.

### Response reliability over extended recording durations is high

For a subset of neurons, the same DMR stimulus was presented a second time (e.g., Supplementary Fig. 2a). Correlations between the resulting STRFs for both large and small PSPs were usually high and significantly exceeded those for spike STRFs (Supplementary Fig. 2b). This fairly high test-retest reliability indicates that the extended recording period required for obtaining STRFs did not compromise the quality of the functional characterization.

### Different mean DMR intensities have only minor effects on STRF

The frequency extent of TRFs is strongly intensity dependent (Fig. 1a, b). We tested for the effects of variations in DMR intensity on STRFs by comparing three sound intensities (38, 54, and 69 dB SPL) in each of five neurons that showed stable recordings over more than 35 min (Supplementary Fig. 3). For all three event types, STRF shape remained quite similar although the STRF magnitude occasionally was reduced at the higher intensity (Supplementary Fig. 3a). Different intensities did not yield significant changes in peak latency (Supplementary Fig. 3b, middle) or STRF bandwidths for individual or double frequency peaks (Supplementary Fig. 3b, right). Thus, broadband stimulation reduces robust intensity effects on frequency selectivity for both sub- and suprathreshold events that are commonly observed for narrowband stimuli, likely due to corticocortical influences^24–26^.

### Double-peaked STRFs with harmonic frequency relationships are more common for PSPs

**Fig. 3:**
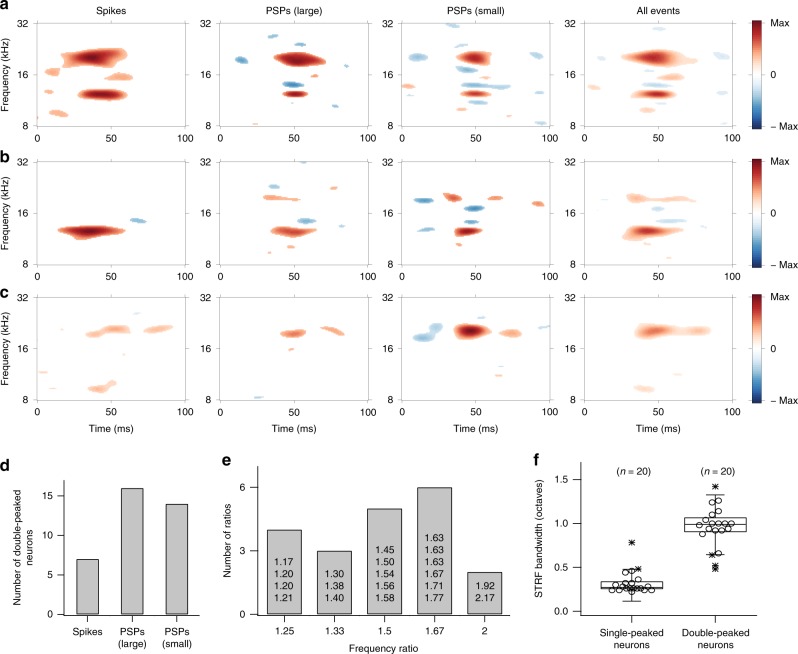
Properties of double-peaked neurons in response to the DMR stimulus. Three representative double-peaked neuron responses (**a**–**c**) are illustrated. Each row was obtained from a different animal. **a** All STRFs showed double-peaked responses at ~12.3 kHz and ~20.0 kHz with a ratio of 1.63 and had a bandwidth of ~0.98 octaves. The number of peaks included in computing each STRF was 1147 for spikes, 660 for large PSPs, 1361 for small PSPs, and all number of peaks for all events. **b** The PSP STRFs showed double-peaked responses at ~12.6 kHz and ~19.4 kHz with a ratio of 1.54 and had a bandwidth of ~0.94 octaves. The number of peaks included in computing each STRF was 1220 for spikes, 1591 for large PSPs, 1215 for small PSPs, and all number of peaks for all events. **c** The spike STRF had double-peaked responses at ~9.3 kHz and ~20.2 kHz with a ratio of 2.17 and a bandwidth of ~1.36 octaves. The number of peaks included in computing each STRF was 1028 for spikes, 424 for large PSPs, 708 for small PSPs, and all number of peaks for all events. **d** Quantification of double-peaked responses. **e** Harmonically related double-peaked responses. Each bar graph shows raw frequency ratios added to counting. Ratios were estimated from STRFs for all events. **f** The bandwidth comparison between single-peaked and double-peaked neurons. STRF bandwidths were estimated from all events. The lower and upper hinges are at the 25 (Q25) and 75 (Q75) percentiles, respectively. The median bandwidth values, the middle line between hinges, were 0.28 for single-peaked neurons and 0.96 for double-peaked neurons. The lower and upper inner fences are set at Q25 − 1.5×(Q75 − Q25) and Q75 + 1.5×(Q75 − Q25), respectively. Outliers beyond inner fences are indicated by asterisks.

Among all recorded neurons, 50% (Fig. 3f; *n* = 20/40) showed single-peaked STRFs while 50% (Fig. 3f; *n* = 20/40) had double-peaked responses in either the spiking or the subthreshold STRFs (Fig. 3a–c). Double-peaked responses in spike STRFs were less common (Fig. 3d; *n* = 7/40; 17.5%). Harmonic relationships between the peaks, i.e., whole-number ratios of a higher frequency to a lower frequency (e.g., 2/1, 3/2, etc.), were common. The observed frequency ratios (*n* = 20) of doubled-peaked STRFs obtained for all event types were assigned to the closest of five whole-number ratio categories (Fig. 3e; i.e., 1.25, 1.33, 1.5, 1.67, and 2). The actual ratios fell within 4.5% of these categories and reflected a wide range of observed frequency ratios, from 5/4 to 2/1. A ratio of 1.67 (i.e., 5/3) was most prevalent (Fig. 3e; *n* = 6/20). This indicates that convergence of low-frequency harmonic components is common in subthreshold activity but is reduced by ~61% in spiking outputs (double-peaked STRFs for all events, *n* = 20/40; double-peaked PSP STRFs, *n* = 18/40; double-peaked spike STRFs, *n* = 7/40).

### STRF modulation properties can differ between subthreshold and spiking events

**Fig. 4:**
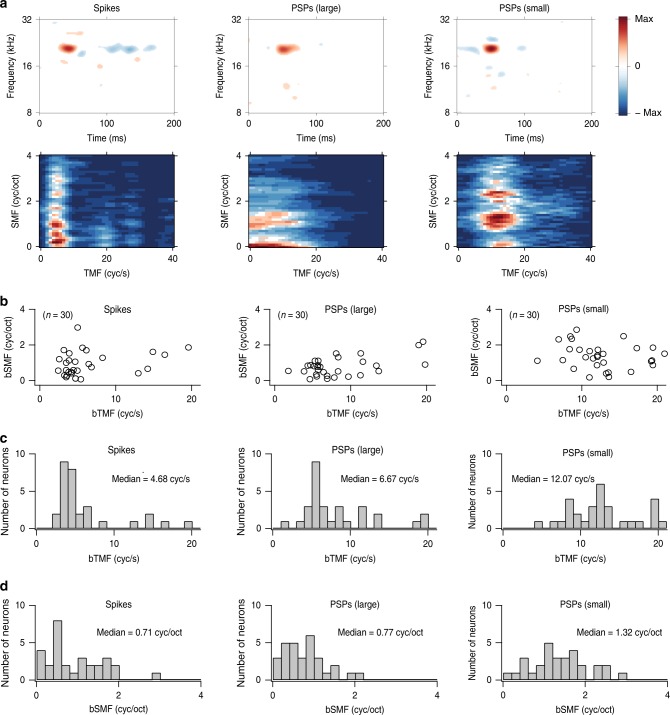
Best modulation frequency. **a** An example of the modulation processing analysis. Top, STRFs’ best frequency was ~20.5 kHz. STRFs were plotted on the same absolute scale. The number of peaks included in computing each STRF was 851 for spikes, 925 for large PSPs, and 1155 for small PSPs. Bottom, the corresponding RTFs are located at the bottom of each STRF. The spectral modulation preferences for the example neuron were 0.56 cyc/oct (spikes), 0.10 cyc/oct (large PSPs), and 1.33 cyc/oct (small PSPs), which also indicate distinct modulation preferences for spiking and subthreshold activity. **b** Plots of bSMFs versus bTMFs for spikes (left), large PSPs (middle), and small PSPs (right) from neurons (*n* = 30/40) with significant STRF subfields for spiking, large PSP, and small PSP events. There were weak or moderate correlations (spikes: Pearson’s *r* = 0.28, *p* = 0.13; large PSPs: Pearson’s *r* = 0.53, *p* = 0.003; small PSPs: Pearson’s *r* = −0.19, *p* = 0.31). **c** Histograms of bTMFs for spikes (left), large PSPs (middle), and small PSPs (right). **d** Histograms of bSMFs for spikes (left), large PSPs (middle), and small PSPs (right).

Because spectral and temporal modulations are inherent features of natural sounds, it is important to understand how modulation information carried by synaptic and spiking activity differs, and how it may be transformed by the spike generation process. STRFs with significant subfields (e.g., Fig. 2a; *n* = 30/40) were transformed into ripple transfer functions (RTFs; see the “Methods” section) that reflect the preferred spectral and temporal modulations present in the stimuli. The spike-based STRFs (Fig. 4a, top left) showed a significant high-energy subfield (red) combined with a longer-latency suppression subfield (blue). By contrast, the STRF for small PSPs showed a short as well as a long-latency suppression subfield indicating potential temporal modulation diversity (Fig. 4a, top right). These STRF differences are reflected in RTF differences (Fig. 4a, bottom row) from the same neuron, which resulted in a best temporal modulation frequency (bTMF) of 4.94 Hz for spikes, versus 6.91 Hz for large PSPs, and 12.07 Hz for small PSPs. The bTMFs were significantly higher for small PSPs compared to spikes (Fig. 4c; Table 1; Δ (difference) = 6.25 ± 5.43 Hz, mean ± s.d., *n* = 30/40) and to large PSPs (Δ = 4.21 ± 5.34 Hz, mean ± s.d., *n* = 30/40). Furthermore, best spectral modulation frequencies (bSMFs) were statistically different between small PSPs and spikes (Fig. 4d; Table 1; Δ = 0.44 ± 0.89 cyc/oct, mean ± s.d., *n* = 30/40) and between small PSPs and large PSPs (Δ = 0.58 ± 0.95 cyc/oct, mean ± s.d., *n* = 30/40).

**Table 1:** Functional properties of spiking and PSP STRFs.

STRF properties	Spikes (mean ± s.d.)	PSPs (large) (mean ± s.d.)	PSPs (small) (mean ± s.d.)	*p*-value
Bandwidth [single bands] (octaves)	0.32 ± 0.12 (*n* = 31)	0.28 ± 0.11 (*n* = 31)		0.1127
	0.32 ± 0.12 (*n* = 31)		0.25 ± 0.07 (*n* = 31)	0.0016**
		0.28 ± 0.11 (*n* = 31)	0.25 ± 0.07 (*n* = 31)	0.0562
Bandwidth [all bands] (octaves)	0.50 ± 0.32 (*n* = 30)	0.57 ± 0.33 (*n* = 30)		0.3758
	0.50 ± 0.32 (*n* = 30)		0.50 ± 0.32 (*n* = 30)	0.9926
		0.57 ± 0.33 (*n* = 30)	0.50 ± 0.32 (*n* = 30)	0.1391
Latency (ms)	42.7 ± 11.1 (*n* = 30)	48.7 ± 11.8 (*n* = 30)		0.0229*
	42.7 ± 11.1 (*n* = 30)		45.2 ± 8.2 (*n* = 30)	0.1874
		48.7 ± 11.8 (*n* = 30)	45.2 ± 8.2 (*n* = 30)	0.0451*
Duration (ms)	43.0 ± 24.1 (*n* = 30)	43.6 ± 18.8 (*n* = 30)		0.8959
	43.0 ± 24.1 (*n* = 30)		34.5 ± 10.3 (*n* = 30)	0.0489*
		43.6 ± 18.8 (*n* = 30)	34.5 ± 10.3 (*n* = 30)	0.0175*
bTMF (cyc/s)	6.35 ± 4.50 (*n* = 30)	8.39 ± 4.69 (*n* = 30)		0.0542
	6.35 ± 4.50 (*n* = 30)		12.60 ± 4.26 (*n* = 30)	7 × 10^−7^**
		8.39 ± 4.69 (*n* = 30)	12.60 ± 4.26 (*n* = 30)	0.0002**
bSMF (cyc/oct)	0.93 ± 0.68 (*n* = 30)	0.78 ± 0.53 (*n* = 30)		0.3574
	0.93 ± 0.68 (*n* = 30)		1.36 ± 0.70 (*n* = 30)	0.0120*
		0.78 ± 0.53 (*n* = 30)	1.36 ± 0.70 (*n* = 30)	0.0021**
Event rate (Hz)	1.31 ± 0.92 (*n* = 30)	1.73 ± 0.72 (*n* = 30)		0.0849
	1.31 ± 0.92 (*n* = 30)		1.52 ± 0.56 (*n* = 30)	0.2966
		1.73 ± 0.72 (*n* = 30)	1.52 ± 0.56 (*n* = 30)	0.2122
Feature selectivity index	0.15 ± 0.08 (*n* = 30)	0.12 ± 0.03 (*n* = 30)		0.0338 *
	0.15 ± 0.08 (*n* = 30)		0.15 ± 0.04 (*n* = 30)	0.7493
		0.12 ± 0.03 (*n* = 30)	0.15 ± 0.04 (*n* = 30)	0.0018**
Nonlinearity threshold, ϴ (s.d.)	0.61 ± 0.87 (*n* = 30)	−0.57 ± 0.87 (*n* = 30)		7 × 10^−6^**
	0.61 ± 0.87 (*n* = 30)		−0.55 ± 0.74 (*n* = 30)	4 × 10^−6^**
		−0.57 ± 0.87 (*n* = 30)	−0.55 ± 0.74 (*n* = 30)	0.9214
Nonlinearity transition, σ (s.d.)	1.25 ± 0.48 (*n* = 30)	1.93 ± 0.67 (*n* = 30)		0.0002**
	1.25 ± 0.48 (*n* = 30)		1.59 ± 0.27 (*n* = 30)	0.0025**
		1.93 ± 0.67 (*n* = 30)	1.59 ± 0.27 (*n* = 30)	0.0188*

Statistical testing was performed using two-tailed paired Student’s *t*-test; significant differences are indicated by *p < 0.05 and **p < 0.005.

Therefore, large and small synaptic events that do not lead to spiking can exhibit different spectral and temporal modulation preferences. However, the modulation preferences of large non-spiking events are similar to the spiking output. This suggests that cellular-level transformations, including thresholding, increase response selectivity.

### Frequency selectivity differences between TRFs and STRFs

**Fig. 5:**
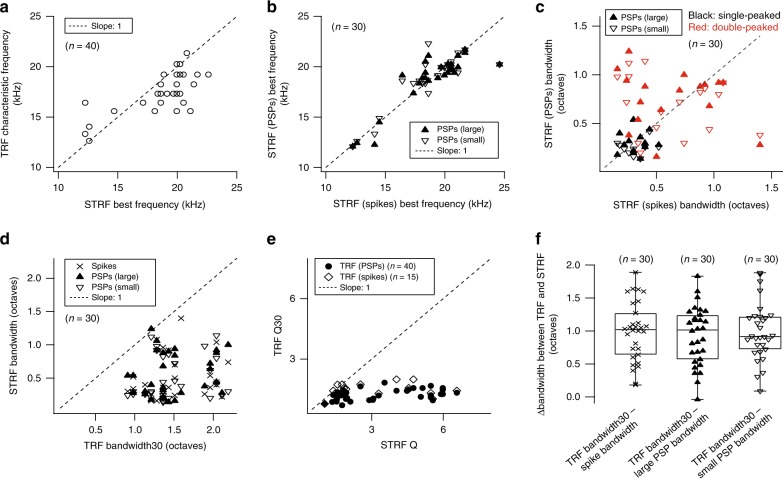
Bandwidth comparison between TRFs and STRFs. **a** Relationship between TRF characteristic frequencies and STRF best frequencies derived from all events (*n* = 40/66 neurons with paired recordings for both, pure tones and DMR stimuli; Pearson’s *r* = 0.73, *p* = 10^−7^). **b** Relationship between spike-based STRF best frequencies and PSP-based STRF best frequencies (*n* = 30/40 neurons with significant STRF subfields for spiking, large PSP, and small PSP events; large PSPs (closed triangles), small PSPs (open triangles)). **c** Relationship between spike-based STRF bandwidths and PSP-based STRF bandwidths (*n* = 30/40 neurons with significant STRF subfields for spiking, large PSP, and small PSP events; large PSPs (closed triangles), small PSPs (open triangles); single-peaked STRFs (black), double-peaked STRFs (red) for spikes, or large PSPs, or small PSPs). Note that red color indicates the presence of a second peak in small PSP-, or large PSP-, or spike-based STRFs. **d** Relationship between STRF bandwidths and TRF bandwidth30s (*n* = 30/40 neurons with significant STRF subfields for spiking, large PSP, and small PSP events). **e** Relationship between TRF Q30s and STRF Qs. The black closed circles indicate the relationship between PSP-based TRF Q30s and STRF Qs derived from all events (*n* = 40/66 neurons with paired recordings for both, pure tones and DMR stimuli; two-tailed paired Student’s *t*-test, *p* = 10^−9^). Open squares indicate the relationship between spike-based TRF Q30s (normalized-driven ratio ≥0.3) and STRF Qs derived from all events (*n* = 15/40 neurons with paired recordings for TRFs (normalized-driven ratio ≥0.3) and STRFs; two-tailed paired Student’s *t*-test, *p* = 0.0025). **f** Bandwidth differences (Δbandwidth) between PSP-based TRFs and STRFs. Δbandwidths (mean ± s.d., *n* = 30/40 neurons with significant STRF subfields for spiking, large PSP, and small PSP events) were 0.99 ± 0.45 octaves (for spikes), 0.92 ± 0.44 octaves (for large PSPs), and 0.99 ± 0.44 octaves (for small PSPs). The median Δbandwidth values, the middle line between hinges, were 1.02 for spikes, 1.01 for large PSPs, and 0.91 for small PSPs. The lower and upper hinges are at the 25th and 75th percentiles. Minimum and maximum values are indicated by whiskers.

The transformation of subthreshold inputs to spiking outputs is a fundamental computational task performed by neurons and may be stimulus-dependent. Thus, we compared the frequency preference and selectivity of sub- and suprathreshold events for tonal and DMR receptive fields. STRF best frequencies and TRF characteristic frequencies were well correlated (Fig. 5a). For STRFs with two best frequencies (Fig. 3), frequencies closest to characteristic frequencies of their corresponding TRFs were chosen for the analysis. Best frequencies from spike-based STRFs were closely matched to STRF-derived estimates for both large and small PSPs (Fig. 5b; *n* = 30/40) with no between-group difference (one-way ANOVA, *p* = 0.99). Therefore, the dominant preferred frequency of neurons is essentially identical for subthreshold and suprathreshold activity, and is independent of the test stimulus (i.e., narrowband versus broadband stimuli).

We next explored whether the range of spectral integration or frequency selectivity of STRFs differs between neuronal PSPs and spikes. We found four general relationships (Fig. 5c): (i) spike-based STRFs with a single frequency band (e.g., Fig. 2a; Fig. 5c, bottom left region) commonly were sharply tuned with bandwidths below ~0.5 octaves (*n* = 20/30; 67%). The PSP-based bandwidths of 65% (*n* = 13/20, large PSPs) and 75% (*n* = 15/20, small PSPs) of these neurons were also below ~0.5 octave; (ii) 30% (*n* = 9/30, large PSPs) and 20% (*n* = 6/30, small PSPs) showed much wider total bandwidths above ~0.5 octaves for PSP-based STRFs largely due to secondary frequency peaks (Fig. 5c, upper left region); (iii) spike-based STRFs with total bandwidths > 0.6 octaves (*n* = 8/30, single or double peaked) had similar bandwidths to the corresponding PSP-based STRFs (Fig. 5c, upper right region); and (iv) one spike-based STRF with two peaks had single-peaked STRFs for both types of PSP events with bandwidths <0.5 octaves (Fig. 5c, bottom right region). Thus, differences in total frequency bandwidth between spiking- and PSP-STRFs are largely due to the emergence or dropping-out of secondary, usually harmonically related frequency components (Fig. 3).

Subthreshold and spiking STRF bandwidths need to be compared to assess whether there is a transformation of the local frequency selectivity, as has been indicated for pure-tone frequency selectivity (Fig. 1f–h). Contrasting individual frequency peaks from spike-based STRFs to their corresponding bandwidths of large PSPs were not statistically different while the bandwidths derived from small PSPs were slightly narrower than for both spike- and large-PSP based STRF peaks (one-way ANOVA, *p* = 0.05) (Table 1). The ~20% higher spectral selectivity of small PSP STRFs is noteworthy although it is not directly reflected in the spiking or large PSP bandwidth.

Comparing the spectral bandwidths obtained with narrowband and broadband stimuli can illuminate the influence of distant frequency components on neuronal frequency integration and selectivity. Most STRF-derived bandwidths were substantially narrower than TRF-derived bandwidths based on both spiking and PSP events (Fig. 5d–f). PSP-based TRF bandwidths were on average ~1 octave wider than total STRF bandwidths for all three STRF event types with no significant group differences (Fig. 5f; one-way ANOVA, *p* = 0.81). Q factors ((best frequency)/bandwidth), another estimate of sharpness of frequency tuning, showed corresponding differences with STRF Q values from all events significantly higher than TRF Q30 values for PSP TRFs and spike TRFs (Fig. 5e).

Overall, the effective spectral integration seen in synaptic responses clearly differed between narrowband and broadband stimuli. The transformational effect of each stimulus type on the corresponding outputs, however, was fairly small. It increased TRF frequency selectivity slightly more than STRF selectivity for individual peaks.

### STRF nonlinearities

**Fig. 6:**
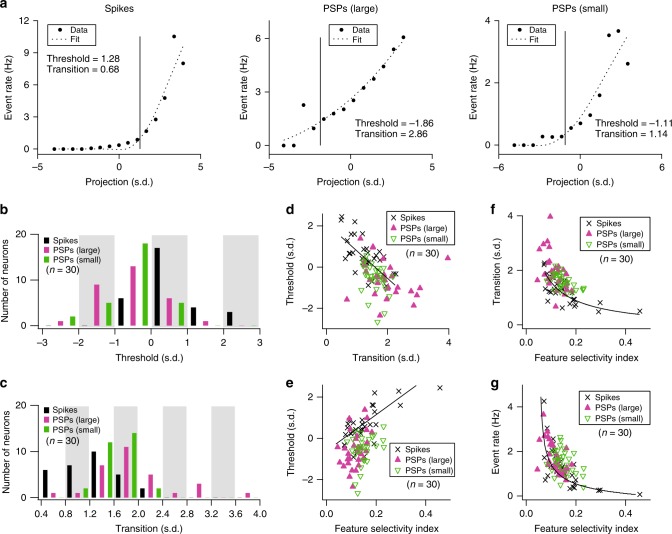
Nonlinearities of STRFs. **a** A representative example illustrates different nonlinearities for spikes and PSPs. **b** Histograms of thresholds (*n* = 30/40 neurons with significant STRF subfields for spiking, large PSP, and small PSP events; Pearson’s *r* = 0.024 and *p* = 0.9 between small PSPs and spikes; Pearson’s *r* = 0.075 and *p* = 0.7 between large PSPs and spikes). The width of a gray bar corresponds to a bin size. Three histograms for spikes, large PSPs, and small PSPs are plotted together in each bin. **c** As in **b**, but for histograms of transitions (*n* = 30/40 neurons with significant STRF subfields for spiking, large PSP, and small PSP events; Pearson’s *r* = −0.007 and *p* = 0.97 between small PSPs and spikes; Pearson’s *r* = −0.078 and *p* = 0.68 between large PSPs and spikes). **d** Relationship between thresholds and transitions (*n* = 30/40 neurons with significant STRF subfields for spiking, large PSP, and small PSP events). **e** Relationship between thresholds and feature selectivity indexes (*n* = 30/40 neurons with significant STRF subfields for spiking, large PSP, and small PSP events; linear fit, Pearson’s *r* = 0.68, *p* = 2 × 10^−5^ for spikes; Pearson’s *r* = 0.39, *p* = 0.0294 for large PSPs; Pearson’s *r* = 0.30, *p* = 0.1045 for small PSPs). **f** Relationship between transitions and feature selectivity indexes (*n* = 30/40 neurons with significant STRF subfields for spiking, large PSP, and small PSP events; power fit, Pearson’s *r* = −0.67, *p* = 4 × 10^−5^ for spikes; Pearson’s *r* = −0.50, *p* = 0.0045 for large PSPs; Pearson’s *r* = −0.54, *p* = 0.0019 for small PSPs). **g** Relationship between event rates and feature selectivity indexes (*n* = 30/40 neurons with significant STRF subfields for spiking, large PSP, and small PSP events; power fit, Pearson’s *r* = −0.63, *p* = 0.0002 for spikes).

In linear-nonlinear filter models of a neuron, the nonlinearity determines the response rate (or probability of an event) as a function of the similarity between the stimulus and a linear filter, which is often modeled by the STRF^27^. The nonlinearity depicts the number of events as a function of the correlation (or projection value) between the stimulus spectrogram preceding an event and the linear filter (STRF). These z-scored projection values are plotted. Nonlinearity characteristics can capture important features of a cell’s input–output transformation (Fig. 6a).

We parametrically described the nonlinearities by fitting an expansive power-law function^19,28,29^. The fitted function has two main parameters: Threshold designates the lowest projection value indicative of a driven response. High thresholds require a close match between stimulus and STRF to achieve a response, corresponding to high feature selectivity; Transition is the smoothness of the nonlinearity transition across threshold. When transition is 0, the function describes hard rectification with little leakage from poorly matched stimuli. High transition values reflect more smoothly varying transitions from absent or weak stimulus/STRF matches to strong matches, indicative of a noisier or leaky thresholding process.

The thresholds of spike nonlinearities were significantly higher than for PSPs (Fig. 6b; Table 1) indicative of a process that transforms noisy synaptic inputs with lower feature-selectivity into less noisy spiking outputs with higher feature selectivity. Additionally, the transition measure for spike nonlinearities is smaller than for either PSP type (Fig. 6c; Table 1). This difference points to a harder rectification process at the spike generation level further reducing the influence of low stimulus/STRF similarities or random events and, thus, enhancing feature selectivity and reducing response variability and contamination. For spikes, nonlinearity threshold and transition covaried (Fig. 6d; Pearson’s *r* = −0.64, *p* = 0.0001) with only weak correlations for either large or small PSPs, not reaching statistical significance.

A direct measure of the degree of feature selectivity is the feature selectivity index (see the “Methods” section). Feature selectivity indexes of 1 indicate that a neuron behaves like a hypothetical feature detector with events occurring only for perfect matches between stimulus and filter, whereas values near 0 indicate that neurons indiscriminately respond to randomly selected stimulus segments. The average feature selectivity index value for spiking events was significantly higher than for large PSPs (Table 1). High feature selectivity index values predict high nonlinearity thresholds, low transitions, and low firing rates. These relationships are clearly expressed for the spiking events (Fig. 6e−g). PSPs indicate similar relationships although the correlations were weaker or not significant.

Higher threshold and lower transition values for spiking versus PSP events signify an essential transformation from active subthreshold information integration to higher, suprathreshold information selection in auditory cortical neurons. Neither the nonlinearity thresholds nor the transition values were correlated between PSPs and spiking events, reflecting that the input and output transformations accomplished by synaptic integration and the spike-generation mechanisms are largely independent from each other and specific to each neuron.

### DMR-evoked excitatory and inhibitory synaptic currents

**Fig. 7:**
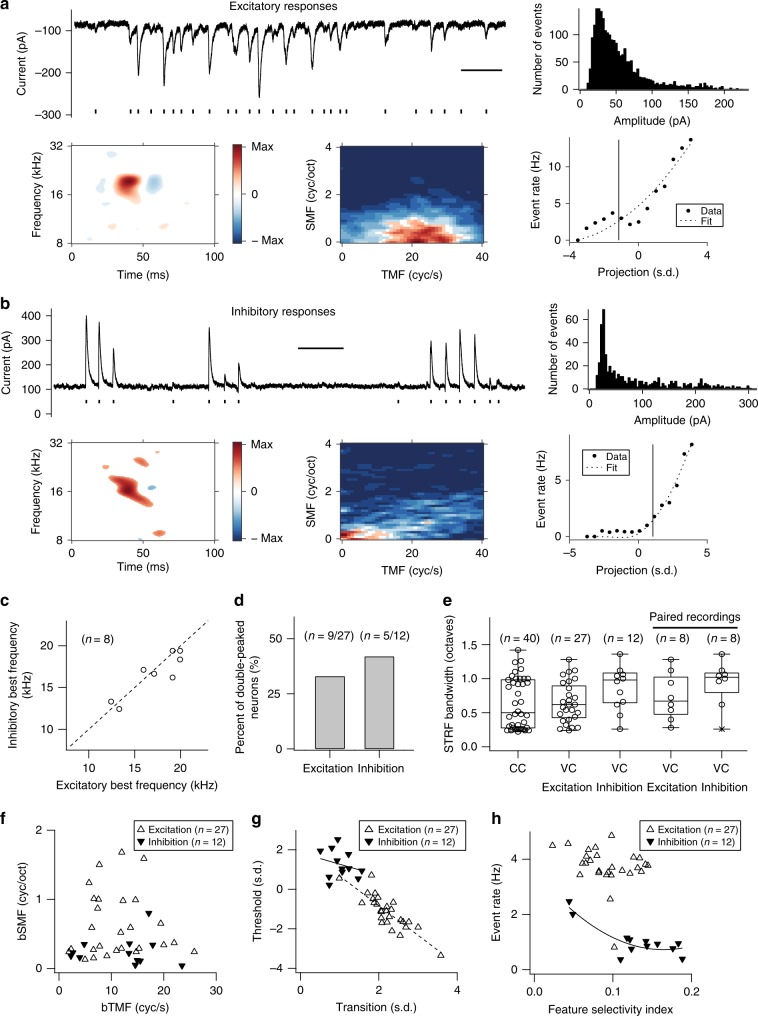
Bandwidths, modulation properties, and nonlinearities of STRFs from in vivo voltage-clamp whole-cell recordings to the DMR stimulus. **a** A representative example of excitation. Top left, a segment of excitatory responses at a holding potential of −70 mV. Scale bar, 0.5 s. The short black line marks below current responses indicate excitatory current peak times. Top right, an amplitude histogram of excitatory currents was obtained from the left recording. Bottom, STRF (left), RTF (center), and nonlinearity (right) for the excitatory responses of this neuron. **b** A representative example of inhibition. Top left, a segment of inhibitory responses at a holding potential of 0 mV. Scale bar, 1 s. The short black line marks below current responses indicate inhibitory current peak times. Top right, an amplitude histogram of inhibitory currents was obtained from the left recording. Bottom, STRF (left), RTF (center), and nonlinearity (right) for the inhibitory responses of this neuron. **c** Relationship between paired inhibitory and excitatory STRF best frequencies (*n* = 8/31 neurons with paired recordings of excitation and inhibition). **d** Quantification of double-peaked responses for excitation (*n* = 9/27) and inhibition (*n* = 5/12). **e** The bandwidth comparison among current-clamp (CC; *n* = 40/66 neurons with paired recordings between pure tones and DMR stimuli) and voltage-clamp (VC (*n* = 31 neurons from 24 mice); excitation (*n* = 27/31), inhibition (*n* = 12/31), paired recordings of excitation and inhibition (*n* = 8/31)) STRFs. The lower and upper hinges are at the 25th and 75th percentiles. The median bandwidth values are indicated by the middle line between hinges. Whiskers indicate minimum and maximum values. Asterisks indicate outliers. **f** bSMFs versus bTMFs for excitation (*n* = 27/31) and inhibition (*n* = 12/31). **g** Relationship between thresholds and transitions for excitation (*n* = 27/31) and inhibition (*n* = 12/31). **h** Relationship between event rates and feature selectivity indexes for excitation (*n* = 27/31) and inhibition (*n* = 12/31).

The observed distinctions in sub- and supra-threshold information processing, particularly reflected in the differences between the derived nonlinearities, raise the question of distinct synaptic contributions. We recorded successfully 31 neurons in voltage-clamp mode and obtained 27 excitatory DMR responses and 12 inhibitory traces with eight neurons yielding both components (e.g., Fig. 7a, b). The amplitude distributions of peak currents (>3 × s.d. above baseline) were unimodal for excitatory postsynaptic currents (EPSCs) and inhibitory postsynaptic currents (IPSCs) and we constructed STRFs across all significant events. For the eight paired recordings, excitatory and inhibitory best frequencies were closely matched (Fig. 7c). Double–peaked STRFs were observed in 35% (Fig. 7d; *n* = 11/31) of the current traces, a similar proportion as for small PSPs (Fig. 3d; *n* = 14/40), and about twice as high as for spiking STRFs (Fig. 3d; *n* = 7/40).

Previous studies with tones suggested that inhibition and excitation are generally co-tuned although slightly broader frequency tuning of the inhibitory inputs was noted^9,13,30,31^. This is also observed here for the STRF bandwidths of EPSCs and IPSCs (Fig. 7e). The inhibitory bandwidth exceeded the excitatory bandwidth by ~27% (Fig. 7e; Table 2). These spectral tuning differences were also reflected in the RTFs by showing lower bSMFs of IPSC versus EPSC STRFs (Fig. 7f). Compared to bSMFs for both spikes and large PSPs, EPSC, and IPSC spectral modulations were lower, especially for the IPSCs. This reflects a sharpening of the spectral bandwidth when combining excitation and inhibition in generating the PSPs.

**Table 2:** Functional properties of excitatory and inhibitory synaptic STRFs.

STRF properties	Excitation (mean ± s.d.)	Inhibition (mean ± s.d.)	Comparison with CC^a^ (mean ± s.d.)	*p*-value
Bandwidth [all bands] (octaves)	0.65 ± 0.30 (*n* = 27)	0.89 ± 0.34 (*n* = 12)		0.0496*
	0.65 ± 0.30 (*n* = 27)		0.64 ± 0.37 (*n* = 40)^b^	0.8738
		0.89 ± 0.34 (*n* = 12)	0.64 ± 0.37 (*n* = 40)^b^	0.0399*
Latency (ms)	35.2 ± 10.9 (*n* = 27)	43.5 ± 14.8 (*n* = 12)		0.0971
Duration (ms)	41.5 ± 16.9 (*n* = 27)	45.6 ± 29.0 (*n* = 12)		0.6591
bTMF (cyc/s)	11.2 ± 6.0 (*n* = 27)	12.0 ± 6.9 (*n* = 12)		0.7132
	11.2 ± 6.0 (*n* = 27)		6.35 ± 4.50 (*n* = 30)^c^	0.0013**
	11.2 ± 6.0 (*n* = 27)		8.39 ± 4.69 (*n* = 30)^d^	0.0599
	11.2 ± 6.0 (*n* = 27)		12.60 ± 4.26 (*n* = 30)^e^	0.3023
		12.0 ± 6.9 (*n* = 12)	6.35 ± 4.5 (*n* = 30)^c^	0.0190*
		12.0 ± 6.9 (*n* = 12)	8.39 ± 4.69 (*n* = 30)^d^	0.1154
		12.0 ± 6.9 (*n* = 12)	12.60 ± 4.26 (*n* = 30)^e^	0.7862
bSMF (cyc/oct)	0.62 ± 0.47 (*n* = 27)	0.25 ± 0.21 (*n* = 12)		0.0013**
	0.62 ± 0.47 (*n* = 27)		0.93 ± 0.68 (*n* = 30)^c^	0.0527
	0.62 ± 0.47 (*n* = 27)		0.78 ± 0.53 (*n* = 30)^d^	0.2453
	0.62 ± 0.47 (*n* = 27)		1.36 ± 0.70 (*n* = 30)^e^	1 × 10^−5^**
		0.25 ± 0.21 (*n* = 12)	0.93 ± 0.68 (*n* = 30)^c^	1 × 10^−5^**
		0.25 ± 0.21 (*n* = 12)	0.78 ± 0.53 (*n* = 30)^d^	3 × 10^−5^**
		0.25 ± 0.21 (*n* = 12)	1.36 ± 0.70 (*n* = 30)^e^	1 × 10^−9^**
Event rate (Hz)	3.72 ± 0.74 (*n* = 27)	1.06 ± 0.61 (*n* = 12)		8 × 10^−12^**
Feature selectivity index	0.09 ± 0.03 (*n* = 27)	0.13 ± 0.05 (*n* = 12)		0.0242*
	0.09 ± 0.03 (*n* = 27)		0.15 ± 0.08 (*n* = 30)^c^	0.0003**
	0.09 ± 0.03 (*n* = 27)		0.12 ± 0.03 (*n* = 30)^d^	0.0096*
	0.09 ± 0.03 (*n* = 27)		0.15 ± 0.04 (*n* = 30)^e^	6 × 10^−8^**
		0.13 ± 0.05 (*n* = 12)	0.15 ± 0.08 (*n* = 30)^c^	0.2126
		0.13 ± 0.05 (*n* = 12)	0.12 ± 0.03 (*n* = 30)^d^	0.3817
		0.13 ± 0.05 (*n* = 12)	0.15 ± 0.04 (*n* = 30)^e^	0.1950
Nonlinearity threshold, ϴ (s.d.)	−1.14 ± 0.87 (*n* = 27)	1.26 ± 0.70 (*n* = 12)		8 × 10^−9^**
	−1.14 ± 0.87 (*n* = 27)		0.61 ± 0.87 (*n* = 30)^c^	4 × 10^−10^**
	−1.14 ± 0.87 (*n* = 27)		−0.57 ± 0.87 (*n* = 30)^d^	0.0175*
	−1.14 ± 0.87 (*n* = 27)		−0.55 ± 0.74 (*n* = 30)^e^	0.0089*
		1.26 ± 0.70 (*n* = 12)	0.61 ± 0.87 (*n* = 30)^c^	0.0190*
		1.26 ± 0.70 (*n* = 12)	−0.57 ± 0.87 (*n* = 30)^d^	1 × 10^−7^**
		1.26 ± 0.70 (*n* = 12)	−0.55 ± 0.74 (*n* = 30)^e^	2 × 10^−7^**
Nonlinearity transition, σ (s.d.)	2.21 ± 0.49 (*n* = 27)	1.03 ± 0.31 (*n* = 12)		2 × 10^−10^**
	2.21 ± 0.49 (*n* = 27)		1.25 ± 0.48 (*n* = 30)^c^	9 × 10^−10^**
	2.21 ± 0.49 (*n* = 27)		1.93 ± 0.67 (*n* = 30)^d^	0.0813
	2.21 ± 0.49 (*n* = 27)		1.59 ± 0.27 (*n* = 30)^e^	1 × 10^−6^**
		1.03 ± 0.31 (*n* = 12)	1.25 ± 0.48 (*n* = 30)^c^	0.0828
		1.03 ± 0.31 (*n* = 12)	1.93 ± 0.67 (*n* = 30)^d^	6 × 10^−7^**
		1.03 ± 0.31 (*n* = 12)	1.59 ± 0.27 (*n* = 30)^e^	3 × 10^−5^**

Statistical testing was performed using two-tailed unpaired Student’s *t*-test; significant differences are indicated by *p  < 0.05 and **p  < 0.005.

^a^Current-clamp (CC) recordings.

^b^STRFs for all events.

^c^STRFs for spikes.

^d^STRFs for large PSPs.

^e^STRFs for small PSPs.

Temporal modulation tuning of EPSCs and IPSCs was closely matched (Table 2). These values were significantly higher than bTMFs for spikes (Table 2), indicating a substantial functional transformation of temporal information by the integration of synaptic inputs.

Nonlinearities of EPSCs and IPSCs reveal essential distinctions in how these two information streams contribute to the information transformation at the synaptic interface. Higher nonlinearity transition values combined with low, generally negative thresholds for the EPSCs result in a soft, noisy rectification, low stimulus selectivity and, consequently, more random events and higher rates of excitatory synaptic inputs. Nonlinearities of the IPSCs had lower transition values and higher thresholds with lower rates signaling a less noisy and more specific inputs compared to the excitatory inputs (Fig. 7g, h). Thresholds for EPSCs were significantly below spikes and PSPs whereas IPSC threshold were well above those found for spikes and PSPs. Transition values for EPSCs were higher than those for spikes and PSPs whereas IPSC transition values were similar to those of spiking events but lower than those for PSPs. Feature selectivity index values were slightly higher for inhibitory inputs compared to excitatory inputs in accordance with the higher nonlinearity thresholds (Table 2). Feature selectivity indexes for IPSCs were not different to those of spikes and PSPs while feature selectivity indexes of EPSCs were smaller than those obtained for spikes and PSPs. Overall, properties of excitatory synaptic nonlinearities corresponded fairly closely to those of the large PSPs while stimulus-related functional aspects, such as spectral and temporal modulation properties, underwent substantial transformations from synaptic to spiking activity.

## Discussion

By contrasting subthreshold and spiking events emanating from distinctly different stimulus classes, tones and DMRs, we made three main observations: (1) Assessment of STRFs of excitatory and inhibitory synaptic currents revealed a much higher response selectivity for inhibitory inputs, a wider spectral bandwidth of inhibitory versus excitatory STRFs, and higher temporal modulation capacities than for high-amplitude PSPs and spiking events. (2) STRFs derived separately for high- and low-amplitude PSPs differed in their temporal preferences, but not in spectral preferences. (3) Spectral tuning assessed with broadband stimuli was substantially sharper than seen with narrowband stimuli for non-spiking PSPs and spiking events. Combined, we characterized a set of stimulus-dependent aspects of integration and transformation between auditory cortical inputs and outputs.

We distinguished between two types of PSP events based on response magnitude. The high similarity of modulation preference between large PSPs and spiking events indicates that the properties of small PSP events do not directly shape the effective functional input–output transformation. The differences in the modulation preferences between large and small PSPs suggest two parallel input pathways and/or synaptic networks serving different synaptic populations. Studies have established the convergence of various thalamo-cortical and cortico-cortical pathways to A1 neurons^26,32,33^. The higher temporal following capacity of the small PSP events might indicate that its main source is thalamic in origin, which often prefers faster amplitude modulation rates and higher spectral modulations than cortical neurons^34^. Two types of synapses have been shown to affect auditory cortical neurons^35^, providing two modes of transmission tuned for specific roles. The low probability synapses showed low success probability, small current amplitudes, a low degree of short-term synaptic depression and higher temporal precision. In contrast, the high probability synapses illustrated high success probability, larger current amplitudes, marked short-term depression and lower temporal precision. It can be speculated that the small PSPs observed here may be driven by the low probability synapses and the large PSPs by the high probability synapses. Since small PSP events, in contrast to large PSPs, prefer higher spectral modulation stimuli, it appears that the convergence of spectral tuning from the two synaptic networks may also differ.

EPSCs did not reveal corresponding bimodal magnitude or temporal modulation distributions but showed a bTMF distribution similar to small PSPs. One potential contribution to the selective reduction of faster temporal modulations for large PSP events could be a higher synchrony between phase-locked excitatory and inhibitory events that is more likely to occur at high temporal modulations. By contrast, responses to low temporal modulations may be accompanied by a timing mismatch of phase-locked excitatory and inhibitory currents, thus failing to effectively suppress excitatory inputs.

The manner (“how”) in which PSPs and spikes are generated substantially differs and is largely reflected in differences in their nonlinearities. PSP events had low thresholds (−0.6 s.d.) and EPSC thresholds were even lower (−1.14 s.d.), resulting in noisy trains with many events that mark stimuli with little similarity to the STRF. In the spiking responses, the distribution of nonlinearity thresholds (θ) was centered at approximately 0.6 s.d., implying that the stimulus-filter similarity had to be sufficiently high for spike rates to be discernible. This mean threshold is slightly below that for spikes in cat auditory cortex (1.5 s.d.)^19^ or monkey visual cortex (~1.0 s.d.)^36^, potentially due to species-specific or anesthesia-related differences. Since the precision of stimulus envelope phase-locking in mouse cortical neurons is usually less than in cats or monkeys^37^, the reduced threshold-values are not unexpected given that STRFs depend on high event-time precision. The surprisingly high nonlinearity thresholds and, consequently, high stimulus selectivity of IPSCs (1.26 s.d.) appears to effectively curtail the transmission of equally well-matched, fast excitatory inputs. It also reflects distinct differences in what drives the excitatory and inhibitory inputs to a neuron. The low noise-level and high feature-selectivity of IPSCs indicates the dominance of a functionally more restricted pathway, such as via parvalbumin-expressing interneurons^22,38,39^. By contrast, the low threshold values of EPSCs contribute some functional aspects only loosely related to the stimulus features reflected in the STRF. Such feature-independent inputs may be the result of the convergence of top-down inputs from higher-order cortical areas or other sensory areas, and may represent activity that encodes for higher-order, state-dependent and context-driven auditory functions including stimulus probabilities, predictive signaling, expectations, motivation, decision-making, memory and other task-related information^40–42^.

Nonlinearity transition values for PSPs were relatively high and also resulted in noisier event trains compared to spikes. EPSC transitions were equal or higher than for PSPs. By contrast, IPSC transitions were even lower than for spikes, again reflecting input trains with low noise contamination. The threshold and transition differences between EPSCs and IPSCs are similar, but more pronounced, to what has been observed for spikes in putative excitatory and inhibitory neurons in cat A1^38^. Both, nonlinearity threshold and transition, were negatively correlated, and control the degree of change in response specificity between input and output. Feature selectivity index was higher for neurons with lower event rates, higher thresholds, and lower transition values (Fig. 6e–g). Spiking events altogether have higher feature selectivity index than large PSPs but not small PSPs. EPSC and IPSC values were similar to those of the PSPs. Thus, both input–output transformations (from PSCs to PSPs to spikes) increase the signal-to-noise ratio and provide improved stimulus-feature selectivity, thereby reducing spurious stimulus-filter matches, and enhancing the ability to detect, transmit and eventually identify signals.

Tonal stimuli are short, onset-heavy and spectrally restricted, whereas DMRs are characterized by their long duration, relative lack of sharp onset features and broad frequency extent. These stimulus classes represent extremes along a continuum of properties observed in natural stimuli. The resulting receptive field differences from these two classes—at least when considering spectral selectivity—bracket the processing attributes that natural stimuli with properties between these special cases will likely undergo. The tonal versus DMR disparities likely arise through differences in convergent corticocortical influences from frequencies away from the neurons’ preferred frequency range. Adaptation from synaptic depression at the thalamocortical and corticocortical synapses, as well as reduced cellular driving force and somatostatin inhibitory interneurons^39^ may also contribute to reduced STRF bandwidths^43^. In addition, long-lasting broadband stimuli may invoke a network-based form of longer-duration lateral suppression that is enabled by slow, ongoing recurrent synaptic activity^44^. Those effects may be less effective for intermittent narrowband stimuli^43,45^ and increase the tuning differences between TRFs and STRFs.

What effects do the transformations between synaptic inputs and spiking outputs have on the spectral content of the information? For tones, PSP bandwidths, reflecting both excitatory and inhibitory contributions, were ~16% broader compared to spike bandwidths. Sharpening of tuning has been reported before, but some quantitative estimates provide a much stronger narrowing, up to 45%^12,31^. The spectral similarity between the different event-type STRFs was generally high although secondary frequency peaks were often missing in the spiking STRFs. The removal of secondary peaks between sub- and suprathreshold responses likely results from the combined influences of adaptation and network suppression. It further reduces weaker responses between more efficacious input frequencies and the thresholding mechanism for spike generation eliminates even stronger secondary peaks. The effect of adaptation and global network influences on frequency selectivity is further reflected in the relative stability of frequency tuning across DMR intensity (Supplementary Fig. 3). This is reminiscent of the relative intensity-independence of tonal response tuning in the presence of notched noise^25^. The actual network functions and circuit characteristics, as well as the synaptic integration mechanisms that lead to these effects, remain to be elucidated in more detail.

## Methods

### Animal preparation

In vivo whole-cell recordings were obtained from neurons in A1 of mice (female C56BL/6 mice, Charles River) between 4 and 11 weeks of age that were housed in standard cages (1–5 mice per cage) for 1–4 weeks. Mice were anaesthetized with ketamine (90 mg/kg) and xylazine (12 mg/kg) by intraperitoneal injection. This mixture was supplemented by one third of the initial dose to maintain the mouse anesthesia. Dexamethasone (5 mg/kg) and atropine (0.1 mg/kg) were administered to reduce brain swelling and bronchial and salivary secretions, respectively. Lidocaine (2 mg/kg) was applied at surgical sites to relieve pain. Artificial tears were applied on the animal eyes. The animal was kept on a feedback-controlled heating pad to maintain the body temperature. The mouse head was fixed by attaching a metal head-frame to the skull with an adhesive material (C&B Metabond, Parkell). After the head-frame was secured, the craniotomy of approximately 2 mm in diameter over A1 was made according to the stereotaxic coordinates of auditory cortical regions^46,47^. The brain surface was covered with 2% agarose in saline after the dura was removed. Before we performed in vivo whole-cell recordings, multiunit recordings with a 1 MΩ tungsten electrode (MicroProbes) were obtained at a depth of ~400 µm to confirm tonotopic progression for A1^47^. All procedures were conducted under the protocol approved by the Institutional Animal Care and Use Committee of the University of California, San Francisco according to the National Institutes of Health guidelines.

### Stimuli

Pure tones and the DMR stimulus were generated using the Matlab (Mathworks) software and were presented to the mouse’s left ear by a calibrated free-field speaker in a sound-shielded anechoic chamber (IAC). Pure tones were comprised a set of 360 combinations of 8 intensities that differed by 10 dB, from 0 to 70 dB SPL, and 45 frequencies equally spaced in the logarithmic scale, from 4 to 40 kHz. The set was presented in random order, and each tone lasted 50 ms. Each tone was followed by 250 ms of deadtime^48^. To analyze TRF bandwidths, the bandwidth at 30 dB (bandwidth30) was measured to be 30 dB above the level that produced the minimum response threshold (i.e., ~0 dB SPL (0 dB SPL (*n* = 63); 10 dB SPL (*n* = 3))). The DMR stimulus^6,49^ spanned 0.5–40 kHz, lasted 10 min, and comprised 316 sinusoidal carriers in random phases. The envelope of each carrier is amplitude modulated as a function of time and frequency. The maximum temporal modulation frequency (TMF) is 40 cyc/s, and the maximum spectral modulation frequency (SMF) is 4 cyc/oct. The TMF and SMF values randomly and smoothly changed over the duration of the DMR. The maximum modulation depth of the spectrotemporal envelope was 40 dB, and the mean intensities of the DMR were 38, 54, and 69 dB SPL. All recordings to the DMR stimulus except Supplementary Fig. 3 were collected with a mean intensity of 54 dB SPL. For the intensity test, where the DMR was presented at multiple intensities to the same neuron (Supplementary Fig. 3), the order in which the different intensity stimuli were applied was switched in the different experiment sets. For the bandwidth comparison between TRFs and STRFs, bandwidths in STRFs obtained at 54 dB SPL were compared to bandwidth30s in TRFs. This comparison was made to match the intensity of a single tone in the TRF stimulus to the intensity of a single carrier tone in the DMR. Because the DMR’s effective SPL at ~54 dB SPL is obtained by adding 316 sinusoidal carriers, each carrier alone contributes ~30 dB SPL (54 dB SPL − 10 × log_10_(316/1) ≈ 30 dB/carrier).

### In vivo whole-cell recording

In vivo whole-cell recordings were made from neurons located ~300–500 μm below the pial surface (i.e., ~layer 4)^50,51^ of the mouse’s right A1 with borosilicate patch electrodes using the blind patching technique^52^. Some neurons in the lower part of layer 3 and the upper part of layer 5 might be included. Before a patch pipette was advanced, an Ag/AgCl reference electrode (E205, Warner Instruments) was placed in the recording well. Patch pipettes in current-clamp recordings were filled with a solution containing (in mM): 130 potassium gluconate, 5 NaCl, 4 Mg-ATP, 0.3 Na-GTP, 10 Na-phosphocreatine, 10 HEPES, 0.3 EGTA, and pH = 7.3 (~295 mOsm). Some pipettes included 0.1% biocytin (B4261, Sigma) to verify recording locations (*n* = 10 trials). Obtaining one clear single neuron labeling as shown in Supplementary Fig. 1 requires, first, successful whole-cell recording without a failure (*n* = 1/10) and, second, discontinuation of additional trials to prevent location uncertainty from labeling other cells. Thus, labeling was minimized in this study in favor of more complete functional characterization. Patch pipettes in voltage-clamp recordings contained (in mM) 130 cesium gluconate, 5 TEA-Cl, 4 Mg-ATP, 0.3 Na-GTP, 10 Na-phosphocreatine, 10 HEPES, 0.3 EGTA, and 3 QX-314, pH = 7.3 (~295 mOsm). When a neuron is clamped to the excitatory reversal potential (~0 mV), inhibitory currents are measured. Likewise, excitatory currents are measured at the inhibitory reversal potential (approximately −80 mV)^53^. Whole-cell patch pipettes had 4–10 MΩ resistances to bath. Pipettes were lowered to a target location at 1–2 µm steps per second using a micromanipulator (MP-285, Sutter Instruments) with positive pressure. When a patch pipette was close to a target location, positive pressure was lowered by mouth. The patch electrode was advanced until resistance was increased. When the current pulse amplitude was decreased by ~50%, positive pressure was released, and the cell-attached configuration was made by a gentle suction by mouth. After the whole-cell configuration was achieved in the voltage-clamp mode by applying suction, the mode was switched to the current-clamp mode for current-clamp recordings. All recordings were obtained using an amplifier (Multiclamp 700B, Molecular Devices), low-pass filtered at 5 kHz, and digitized at 10 kHz (DigiData, Molecular Devices) with pClamp 10 software. It typically required ~2 min to estimate a TRF and ~10 min to estimate a STRF. The order to apply pure tones and the DMR stimulus was switched in different experiments. In a current-clamp experiment, the membrane potential was not corrected for liquid junction potential, and no current injections were made. Only recordings where the membrane potential remained stable throughout the recording were analyzed. Data with resting membrane potentials above −50 mV were not included. Series resistance was 20–110 MΩ. In the case that patch pipettes included 0.1% biocytin (Supplementary Fig. 1), the animal was transcardially perfused using 0.1 M PBS with heparin (10 U/ml) followed by 4% paraformaldehyde (PFA). The brain was immersed in 4% PFA for 20 h and then placed into 30% sucrose in PBS until it sank. Brain slices with the thickness of 80 µm were mounted with an antifade mountant (P36970, Invitrogen). Recording locations marked by biocytin were visualized by streptavidin (016-580-084, Jackson ImmunoResearch). Images were acquired on CSU-W1 spinning disk confocal (Nikon Instruments Inc.) and processed using ImageJ public domain software.

### Data analysis

Data were analyzed using Matlab (MathWorks) and IgorPro (Wavemetrics). In the analysis with pure tones, responses in TRFs were determined by the onset latency (5–50 ms) and the PSP amplitude (above 4 × the standard deviation of baseline segments). The normalized-driven ratio was defined by the ratio of the number of spikes to the number of PSPs within a TRF region. Only one spike with one tone was counted when there were bursts in spiking responses. TRFs were obtained by a single trial of the pure tone combination. The characteristic frequency was defined as the frequency having a response at the lowest intensity among frequencies with responses. In the analysis of the DMR responses, the spike-triggered average analysis was used to calculate the STRF. Spikes and amplitude peaks of subthreshold membrane potential responses were detected using the peakfinder Matlab function written by Nathanael Yoder. The peak finding algorithm is based on local maxima using the first and second derivatives and the amplitude difference to nearby minima (~3 mV for current-clamp recordings; ~20 − 40 pA for voltage-clamp recordings). Thus, all the events used the same absolute baseline. It can be interpreted that the large amplitude of large PSPs, compared to small PSPs, results from a prominent increase in neuronal excitability, which is generated by network activity^54^. By discretizing the event time of PSPs and PSCs, standard event-triggered averaging can be used, without concern about the actual event waveform. Significant features of the STRF were extracted by setting a threshold of *p* < 0.05 relative to the shuffled STRFs. To calculate shuffled STRFs, event trains were circularly shuffled at regular intervals of N/niter, where N is the total number of bins in each event train and niter is the number of shuffle iterations per event train. An STRF for each shuffled event train was then calculated and the pixels for all shuffled STRFs formed the null distribution from which a threshold was set for significant pixels in STRFs calculated from real event trains (*p* < 0.05). This method preserves the inter-event interval of the original event train but breaks the correlation between events and the stimulus to generate a null distribution of STRF pixels that can be expected by chance given the EPSP/spike rate of a single neuron. Additionally, a threshold of *p* < 0.01 was tested as well for the bandwidth analysis but did not affect bandwidths in significant subfields of STRFs. The best frequency was determined as the frequency having the strongest response in the STRF obtained from the DMR stimulus at a given intensity. Modulation properties were examined by calculating the two-dimensional Fourier transform of the STRF, which results in the RTF. RTFs show the spectral and temporal relationship of significant and suppressed STRF subfields using spectral and temporal modulation frequencies. bSMFs and bTMFs were peak values (for bandpass) or the mean between the zero and the 3 dB-high side cutoff (for lowpass) from the spectral and temporal modulation transfer functions, obtained by summing the RTF across temporal and SMF, respectively^19^. The nonlinearities were calculated and parameterized following our previous approach^19^. Each stimulus segment, s, that preceded a spike was correlated with the STRF by projecting it onto the STRF via the inner product z = s•STRF. These projections form the probability distribution *P*(z|event). We then formed the prior probability distribution, *P*(z), by projecting a large number of randomly selected stimulus segments onto the STRF. We next calculated the mean and s.d. of *P*(z), μ, and σ. *P*(z|event) and *P*(z) were transformed to units of s.d. via x = (z –μ)/σ to obtain the distributions *P*(z|event) and *P*(x). The nonlinearity is then derived by: *P*(event|z) = *P*(event) *P*(z|event)/*P*(z).

To determine the stimulus selectivity of each event type, we calculated a feature selectivity index^19^. For each event generated by the neuron, the DMR envelope that preceded the event was correlated with the event-specific STRF and a similarity index, SI and its probability distribution *P*(SI) was obtained^19^. The probability distribution of a random selection of stimuli was obtained as well: *Prand*(SI). For each SI probability distribution, the cumulative distribution function was then calculated and the difference between the random and driven event trains was quantified by obtaining the areas, A and Arand, under each cumulative distribution function, from which we then calculated feature selectivity index = (Arand−A)/Arand. Feature selectivity index values vary between 0 and 1, where 0 corresponds to similar distributions for *Prand*(SI) and *P*(SI), i.e., a neuron that responds indiscriminately to stimulus segments, and 1 corresponds to a neuron that is responsive to a very restricted and fixed range of stimulus features.

Response reliability was assessed by computing the correlation coefficients between two STRFs. Harmonic relationships between dominant frequency peaks were calculated as a ratio of the higher frequency divided by a lower frequency. Percent error was calculated by: |(experimental ratio (mean value)−theoretical ratio)/theoretical ratio| × 100%.

Following previous approaches^15^ we used the subthreshold response to estimate STRFs. Since our initial analyses revealed that there were subthreshold amplitude fluctuations whose effects were masked by using the complete subthreshold response without regard to the size of the amplitude fluctuation, we analyzed different types of events that were based on the amplitude of the subthreshold response. This approach is similar to the approach of Machens et al.^15^, since using any sampled recording trace implicitly assumes that the response at each point in time is an event. However, this approach differs from Machens et al.^15^ because it further refines the types of events that are examined and does not weight an event by the size of the response signal. By considering small and large events separately, we mitigated the signal masking that might occur if the complete amplitude range was used. Further, since we used events, we were able to apply, in a straightforward manner, classical event-triggered averaging techniques. Additionally, since the stimulus that we employed was globally uncorrelated, we were able to make unbiased receptive field estimates.

### Reporting summary

Further information on research design is available in the Nature Research Reporting Summary linked to this article.

## Supplementary information

Supplementary Information

Reporting Summary

## Data Availability

All the data supporting the findings of this study are publicly available at the Collaborative Research in Computational Neuroscience data sharing website under DOI citation 10.6080/K07H1GSS.
