# Supplementary material for: Stimulus dependent transformations between synaptic and spiking receptive fields in auditory cortex
Source: Nat Commun. 2020 Feb 27;11:1102. doi: 10.1038/s41467-020-14835-7 (PMC7046699; doi:10.1038/s41467-020-14835-7)
Supplement: Supplementary file 1 — Supplementary Information [file 41467_2020_14835_MOESM1_ESM.pdf]

Stimulus dependent transformations between synaptic and spiking receptive fields in auditory cortex

Kim et al

SUPPLEMENTAL MATERIALS

Stimulus dependent transformations between synaptic and spiking receptive fields in auditory cortex

Authors: Kyunghee X. Kim[1*], Craig A. Atencio[1], Christoph E. Schreiner[1,2]

Affiliations: [1]Coleman Memorial Laboratory, Department of Otolaryngology - Head and Neck Surgery, University of California San Francisco, San Francisco, USA. [2]Center for Integrative Neuroscience, University of California San Francisco, San Francisco, USA.

SUPPLEMENTARY FIGURES

Supplementary Figure 1 | Biocytin labeling of a pyramidal cell from a 25-minute recording

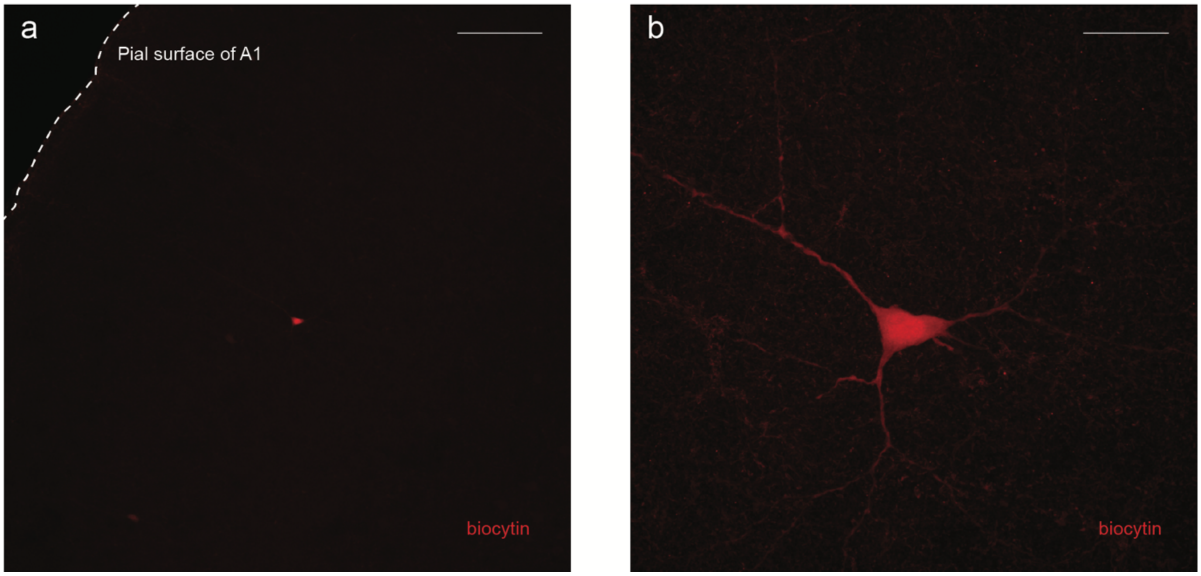

This labeling was acquired from the same neuron as the whole-cell recording in Fig. 3b. Note that this does not definitively identify a specific cell type because of the blind approach applied in this study. Maximum intensity projection was made. (a) A recorded neuron is shown in red (biocytin). Scale bar, 100 μm. (b) An enlarged image of the same neuron as shown in (a). Scale bar, 20 μm.

Supplementary Figure 2 | Reliability of STRFs in response to the DMR stimulus

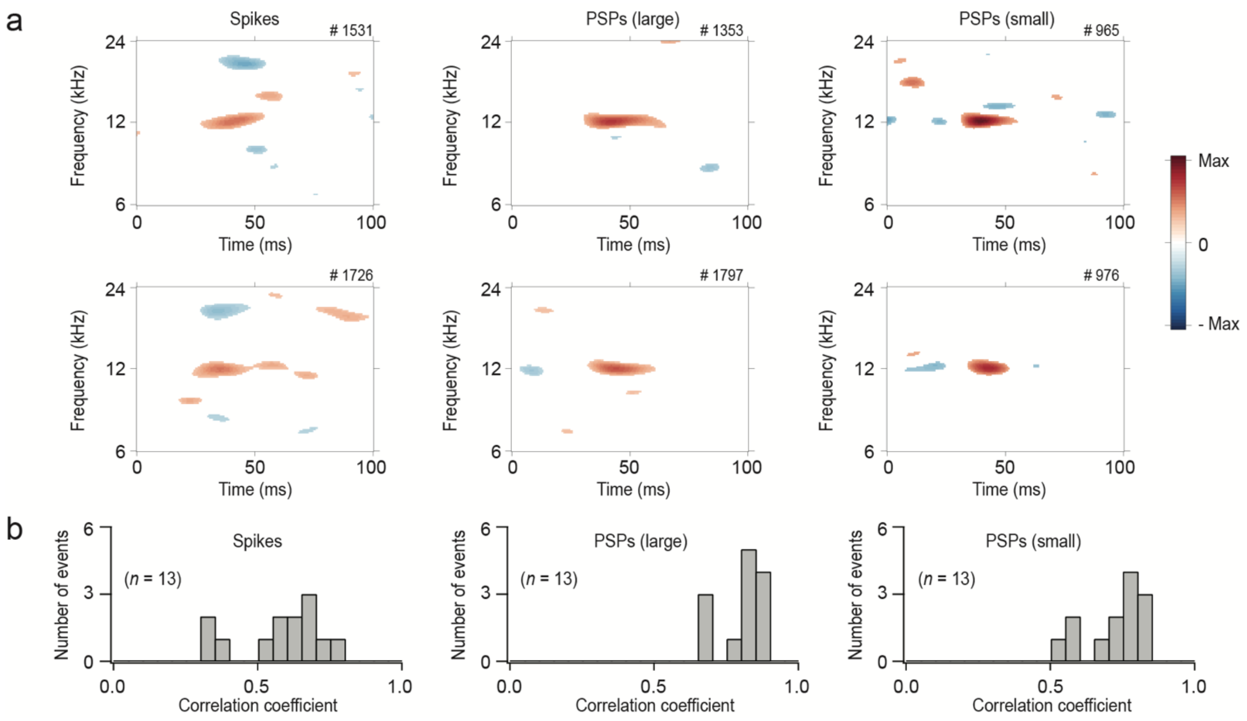

The 13 pairs were from 2 neurons from two different mice: 3 and 5 repetitive recordings to the DMR stimulus generated 3 and 10 pairs, respectively, for testing the similarity between STRFs. (a) A representative example shows STRFs (top and bottom) obtained from the same whole-cell recording, which were estimated on the same absolute scale. The number of peaks included in computing each STRF is indicated in the top right of each STRF. Top, STRFs were obtained from the initial recording to the DMR stimulus. Bottom, STRFs were obtained again using the same DMR stimulus and were comparable to STRFs shown in the top. The neuron's best frequency (top and bottom) was ~12.3 kHz. The correlation coefficient values for this example were 0.30 for spikes, 0.67 for large PSPs, and 0.59 for small PSPs. (b) The similarity between STRFs was assessed using the Pearson correlation coefficient, which ranged from 0.30 to 0.76 for spikes (left), from 0.67 to 0.88 for large PSPs (middle), and from 0.55 to 0.83 for small PSPs (right). Each distribution was shifted away from 0 (two-tailed paired Student's *t*-test, $p = 8 \times 10^{-9}$ for spikes, $p = 7 \times 10^{-14}$ for large PSPs, and $p = 2 \times 10^{-12}$ for small PSPs).

Supplementary Figure 3 | The effect of the DMR stimulus intensity

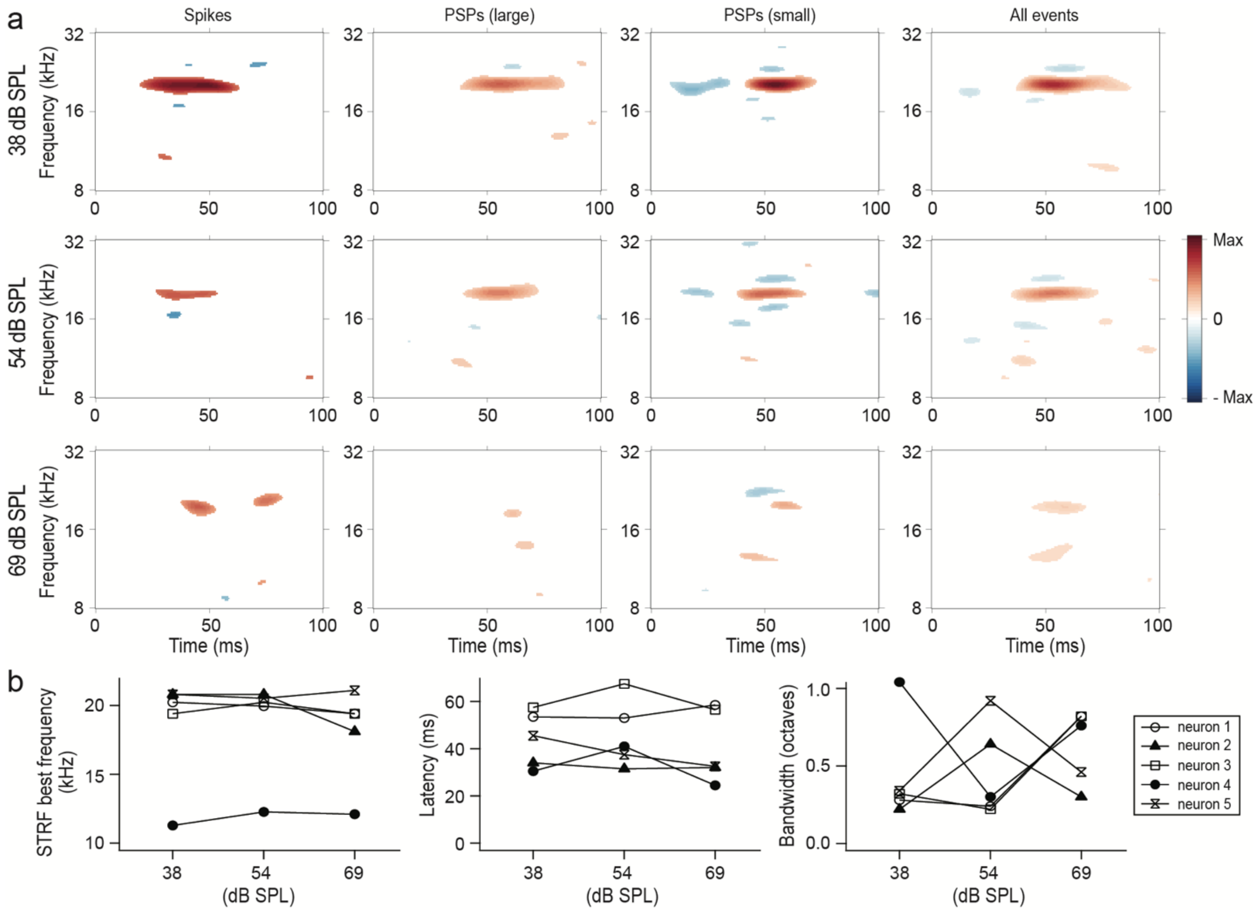

(a) STRFs (top, middle, bottom) resulted from the same neuron and were estimated on the same absolute scale. Top, the mean intensity of the DMR stimulus was set at 38 dB SPL. The number of peaks included in computing each STRF was 203 for spikes, 940 for large PSPs, 755 for small PSPs, and all number of peaks for all events. STRFs had a peak frequency of ~20.2 kHz and a bandwidth of ~0.26 octaves. Middle, the mean intensity was at 54 dB SPL. The number of peaks included in computing each STRF was 200 for spikes, 1,121 for large PSPs, 838 for small PSPs, and all number of peaks for all events. STRFs had a peak frequency of ~20.0 kHz and a bandwidth of ~0.22 octaves. Bottom, the mean intensity was at 69 dB SPL. The number of peaks included in computing each STRF was 347 for spikes, 991 for large PSPs, 873 for small PSPs, and all number of peaks for all events. STRFs for small PSPs and for all events had two frequencies of ~12.8 kHz and ~19.4 kHz with a ratio of 1.5 and a bandwidth of ~0.80 octaves. (b) From STRFs for all events, there was no between-group difference in best frequencies (one-way ANOVA, $p = 0.95$), the average latencies (one-way ANOVA, $p = 0.83$), and STRF bandwidths (one-way ANOVA, $p = 0.56$) with different intensities.
