# Supplementary material for: Stimulus dependent transformations between synaptic and spiking receptive fields in auditory cortex
Source: Nat Commun. 2020 Feb 27;11:1102. doi: 10.1038/s41467-020-14835-7 (PMC7046699; doi:10.1038/s41467-020-14835-7)
Supplement: Supplementary file 2 — Reporting Summary [file 41467_2020_14835_MOESM2_ESM.pdf]

nature research

Corresponding author(s): Kyunghee X. Kim

Last updated by author(s): Jan 25, 2020

Reporting Summary

Nature Research wishes to improve the reproducibility of the work that we publish. This form provides structure for consistency and transparency in reporting. For further information on Nature Research policies, see Authors & Referees and the Editorial Policy Checklist .

Statistics

For all statistical analyses, confirm that the following items are present in the figure legend, table legend, main text, or Methods section.

n/a	Confirmed	
☐	☒	The exact sample size (n) for each experimental group/condition, given as a discrete number and unit of measurement
☐	☒	A statement on whether measurements were taken from distinct samples or whether the same sample was measured repeatedly
☐	☒	The statistical test(s) used AND whether they are one- or two-sided<br>*Only common tests should be described solely by name; describe more complex techniques in the Methods section.*
☐	☒	A description of all covariates tested
☐	☒	A description of any assumptions or corrections, such as tests of normality and adjustment for multiple comparisons
☐	☒	A full description of the statistical parameters including central tendency (e.g. means) or other basic estimates (e.g. regression coefficient) AND variation (e.g. standard deviation) or associated estimates of uncertainty (e.g. confidence intervals)
☐	☒	For null hypothesis testing, the test statistic (e.g. F, t, r) with confidence intervals, effect sizes, degrees of freedom and P value noted<br>*Give P values as exact values whenever suitable.*
☒	☐	For Bayesian analysis, information on the choice of priors and Markov chain Monte Carlo settings
☒	☐	For hierarchical and complex designs, identification of the appropriate level for tests and full reporting of outcomes
☐	☒	Estimates of effect sizes (e.g. Cohen's d, Pearson's r), indicating how they were calculated

Our web collection on statistics for biologists contains articles on many of the points above.

Software and code

Policy information about availability of computer code

Data collection	Electrophysiological recordings were collected using pClamp 10 software. Pure tones and the DMR stimulus that were generated using the Matlab were presented to animals. Images were acquired using Micro-Manager.
Data analysis	Electrophysiological recordings were analyzed using Matlab (MathWorks) and IgorPro (Wavemetrics). Images were processed using NIH ImageJ.

For manuscripts utilizing custom algorithms or software that are central to the research but not yet described in published literature, software must be made available to editors/reviewers. We strongly encourage code deposition in a community repository (e.g. GitHub). See the Nature Research guidelines for submitting code & software for further information.

Data

Policy information about availability of data

All manuscripts must include a data availability statement. This statement should provide the following information, where applicable:

- Accession codes, unique identifiers, or web links for publicly available datasets
- A list of figures that have associated raw data
- A description of any restrictions on data availability

All the data supporting the findings of this study are publicly available at the Collaborative Research in Computational Neuroscience data sharing website under DOI citation http://dx.doi.org/10.6080/K07H1GSS.

Field-specific reporting

Please select the one below that is the best fit for your research. If you are not sure, read the appropriate sections before making your selection.

Life sciences study design

All studies must disclose on these points even when the disclosure is negative.

Sample size	The sample size of mice (65 mice) used in this study is greater than that in a previous study (20 mice) in the auditory field (Tan AY, Wehr M. Balanced tone-evoked synaptic excitation and inhibition in mouse auditory cortex. Neuroscience. 2009 Nov 10;163(4):1302-15) that applied pure tones, because we examined both current-clamp and voltage-clamp recordings using both pure tones and complex sounds, which requires the holding of a neuron for a long period of time.
Data exclusions	We included neurons for analysis when the resting membrane potential of a neuron was below −50 mV in current-clamp mode and when initial series resistance was maintained between 20 and 110 Mohm throughout the recording in voltage-clamp mode, which has similar criteria to mouse studies in the auditory field (Tan AY, Wehr M. Balanced tone-evoked synaptic excitation and inhibition in mouse auditory cortex. Neuroscience. 2009 Nov 10;163(4):1302-15).
Replication	All experiments have been independently replicated from 97 neurons from 65 mice (current-clamp recordings from 41 mice; voltage-clamp recordings from 24 mice) in the attempts of 281 mice.
Randomization	Our study did not contain experimental conditions (control vs experimental groups). So, no randomization was used.
Blinding	Our study did not contain experimental conditions (control vs experimental groups). So, no blinding was used.

Reporting for specific materials, systems and methods

We require information from authors about some types of materials, experimental systems and methods used in many studies. Here, indicate whether each material, system or method listed is relevant to your study. If you are not sure if a list item applies to your research, read the appropriate section before selecting a response.

Materials & experimental systems

n/a	Involved in the study	
☒	☐	Antibodies
☒	☐	Eukaryotic cell lines
☒	☐	Palaeontology
☐	☒	Animals and other organisms
☒	☐	Human research participants
☒	☐	Clinical data

Methods

n/a	Involved in the study	
☒	☐	ChIP-seq
☒	☐	Flow cytometry
☒	☐	MRI-based neuroimaging

Animals and other organisms

Policy information about studies involving animals; ARRIVE guidelines recommended for reporting animal research

Laboratory animals	Mice (female C57BL6) between 4 and 11 weeks of age were purchased from Charles River and were housed in standard cages (1–5 mice per cage) for 1–4 weeks.
Wild animals	The study did not involve wild animals.
Field-collected samples	The study did not involve field-collected samples.
Ethics oversight	All procedures were conducted under the protocol approved by the Institutional Animal Care and Use Committee of the University of California, San Francisco according to the National Institute of Health guidelines.

Note that full information on the approval of the study protocol must also be provided in the manuscript.
